# Towards a New Classification of Cardiomyopathies

**DOI:** 10.1007/s11886-023-01849-y

**Published:** 2023-02-28

**Authors:** Perry Elliott

**Affiliations:** Institute of Cardiovascular Science, University College London, Rayne Institute, 5 University St., London, WC1E 6JF UK

**Keywords:** Classification, Cardiomyopathy, Aetiology, Molecular, Genetic, Nosology

## Abstract

**Purpose of Review:**

The aim of this paper is to briefly summarise the clinical approach to disease notation for cardiomyopathies and to highlight its limitations with respect to the integration of new knowledge about aetiology.

**Recent Findings:**

The paper uses the recently advocated concept of arrhythmogenic cardiomyopathy as an example of the limitations of current classification systems.

**Summary:**

At present, there is no single classification system that meets the needs of all potential users, whether they are basic scientists, clinicians, patients or families. The classical cardiomyopathy subtypes still have utility, but future disease notation needs to be modified to take into account the new and more complete phenotypes and aetiologies.

## Introduction

In medicine, classification systems are used to standardise the nomenclature of disease, often grouping disorders on the basis of shared phenotypes or particular biochemical and genetic characteristics. For more than 50 years, the term cardiomyopathy has been used to denote disorders of the heart muscle that are unexplained by coronary disease or abnormal loading conditions, and subtypes have been defined using relatively simple morphological and physiological parameters. Advances in our understanding of the pathophysiology of heart muscle disease and the development of new aetiology driven therapies are exposing some of the limitations of this traditional approach to disease classification, and in this article, I give a personal perspective on the direction of travel in disease notation.

## The History of the Cardiomyopathy Concept

In 1957, it was Wallace Brigden, a cardiologist working in London, who said that when describing heart muscle disorders, “adjectives such as isolated, idiopathic, non-specific, specific, interstitial, diffuse, and circumscribed abound in the literature; others, such as acute, subacute, chronic pernicious, and malignant, relate to the clinical picture; while still others, such as eosinophilic, allergic, idiosyncratic, and granulomatous hint at aetiology, as does familial cardiomegaly” [1]. He suggested that these imprecise and often inaccurate terms should be replaced by the term cardiomyopathy, which he defined simply as “isolated non-coronary myocardial disease”.

In 1961, Goodwin et al. refined this concept by defining cardiomyopathies as disorders of heart muscle “of unknown or obscure aetiology, often with endocardial, and sometimes with pericardial involvement, but not atherosclerotic in origin” [2]; subtypes of cardiomyopathy were defined using specific morphological and physiological features such as left ventricular hypertrophy and dilatation [3]. While Goodwin et al. recognised that this purely descriptive approach to nomenclature was necessarily limited and probably temporary due to lack of knowledge about the cause of most heart muscle diseases, the morphological categorization of cardiomyopathy has remained largely unchanged to the present day with only minor adjustments including the recognition of a new entity, arrhythmogenic right ventricular cardiomyopathy (ARVC) [4] and a more explicit emphasis on the existence of genetic and non-genetic forms of disease [5].

## Limitations of the Traditional Classification System

Current cardiomyopathy sub-types are defined mostly by a small number of clinical traits such as ventricular wall thickness or function. While still useful as a starting point in the diagnostic pathway, this approach is limited when trying to describe the evolving nature of cardiomyopathies or aetiological complexity. It can also lead to some confusion when analysing disease expression in families which can vary between individuals.

Arrhythmogenic right ventricular cardiomyopathy (ARVC) provides a useful case study for the growing tension between phenotypical and aetiological approaches to disease notation. For decades, ARVC has been defined using consensus criteria that rely on an analysis of right ventricular function (global or regional), histological abnormalities in the form of fibro-fatty replacement of cardiomyocytes, electrocardiographic characteristics, ventricular arrhythmia of right ventricular origin and the presence of familial disease and/or pathogenic variants in desmosomal protein genes [6–8]. While this approach to diagnosis would seem to be the epitome of a disease nomenclature that integrates aetiology with a multiparametric description of phenotype, it also reflects the challenge of merging a historical legacy with new discoveries into a single conceptual framework. Initially, ARVC was recognised solely by the presence of severe right ventricular disease and malignant ventricular arrhythmia, but it very rapidly evolved to encompass a broader phenotype that includes concealed or subclinical phenotypes and, more recently, biventricular disease [9, 10]. The coexistence of left ventricular disease in particular has led to the emergence of a plethora of new terms including arrhythmogenic left ventricular cardiomyopathy, left and right dominant cardiomyopathy, arrhythmogenic dilated cardiomyopathy and, most recently, the catch-all term, arrhythmogenic cardiomyopathy (ACM) which has been used to describe a broad spectrum of aetiologically distinct myocardial diseases defined by the occurrence of potential for ventricular arrhythmia [11•, 12•]. While there have been a number of attempts to define ACM more precisely, a satisfactory universal definition remains elusive, and the term can hamper rational therapeutic decision-making.

## Personalised Medicine as a Driver of Change

Healthcare is moving rapidly towards models based on personalised or stratified medicine in which emergent diagnostic technologies, molecular biology, big data and real time monitoring are used to better target therapies and improve health, social outcomes and cost efficiency. New scientific disciplines such as genomics, transcriptomics, proteomics and metabolomics are essential building blocks of personalised medicine as they provide data that can be used to separate patients into specific groups amenable to tailored therapy at an earlier stage than is currently possible. However, the potential of these disciplines to transform human health can only be realised by integrating biological data into disease models that reflect the complex phenotypes seen in clinical practice [13].

Over the past 50 years, our understanding of the pathogenesis of cardiomyopathies has been transformed, and it is clear that the small number of clinical phenotypes (hypertrophic, dilated etc.) encompass a much broader spectrum of disease which is determined by rare and common genetic variation, environmental triggers, ageing and comorbidity. In the current ESC classification scheme for cardiomyopathies [5], there is a nod to aetiology in the tacit recognition of genetic/familial versus non-genetic/familial causes of disease, but this only scratches the surface of the aetiological complexity underlying all cardiomyopathy phenotypes.

For a couple of decades, the value of a new classification system based on the underlying pathophysiology rather than the gross morphology and function of the heart has been debated periodically. Given that many heart muscle disorders are caused by mutations in genes that encode cardiac proteins, it is logical to consider a nosology that defines diseases according to the causative molecular abnormality, for example, diseases of sarcomeric, cytoskeletal and nuclear envelope proteins. The argument against this approach (mea culpa) has been that the pathway from diagnosis to treatment rarely begins with the identification of an underlying genetic defect but starts instead with a symptomatic presentation or the incidental finding of clinical signs or abnormal tests. However, this argument may be less tenable in the present era as the widespread use of genetic testing and other advanced diagnostic tests leads to new clinical scenarios in which the traditional cardiomyopathy phenotypes no longer apply.

## MOGES System for Disease Notation

**Fig. 1:**
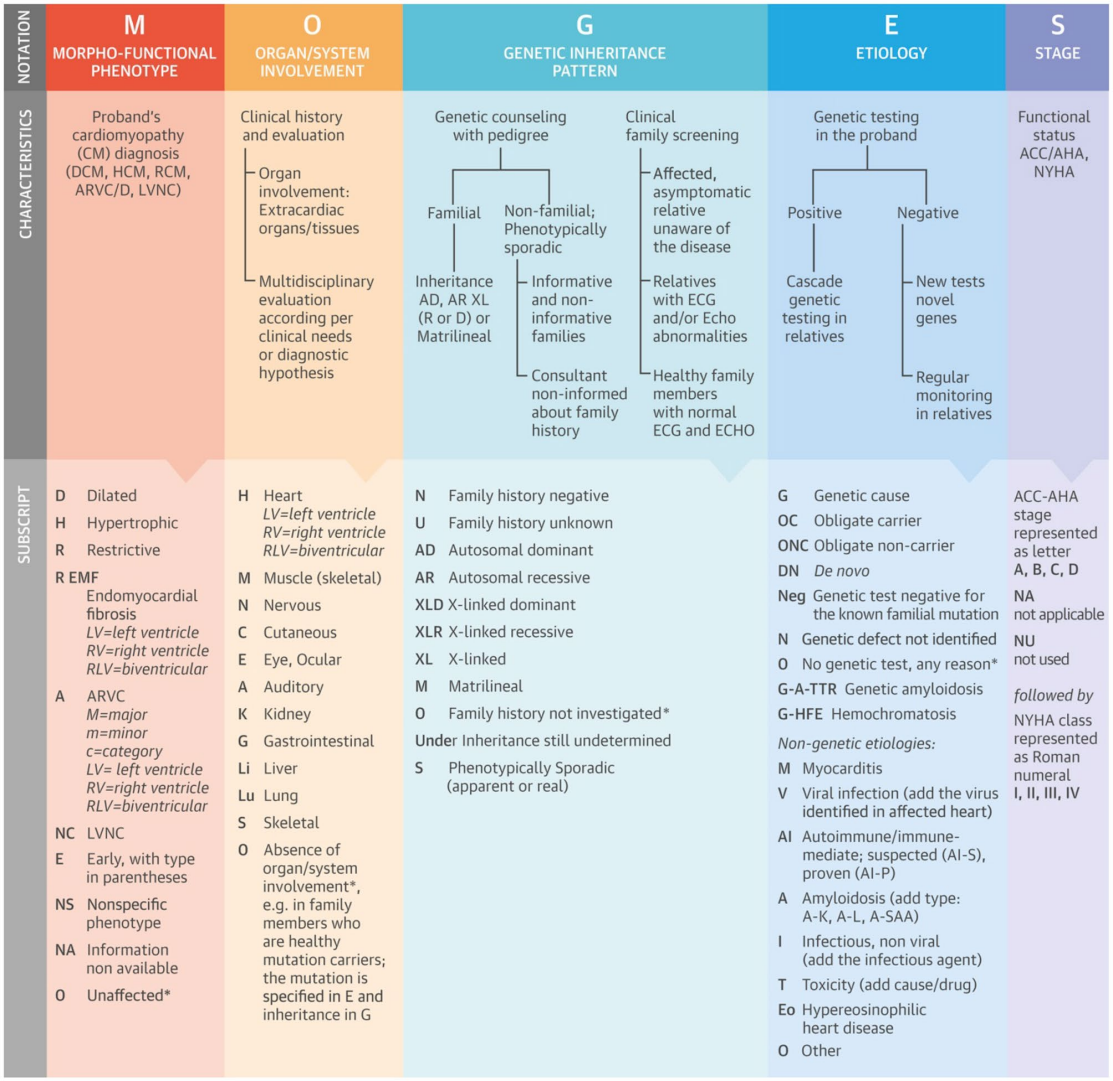
The MOGES nosology system for classifying cardiomyopathies. (M) Morphofunctional phenotype. This description is designed to contain additional information. These red flags are placed in parentheses after the notation of morphofunctional phenotype. (O) Organ/system involvement. This described involvement of other organs. (E) Aetiologic annotation provides information on specific disease genes and mutation, as well as a description of nongenetic causes of disease. (G) May denote a genetic disease, supporting family monitoring strategies. (S) Stage. This provides a simple description of functional limitation using existing scores such as the New York Heart Association class. Key: ACC, American College of Cardiology; AHA, American Heart Association; ARVC/D, arrhythmogenic right ventricular cardiomyopathy/dysplasia; DCM, dilated cardiomyopathy; ECG, electrocardiogram; ECHO, echocardiogram; HCM, hypertrophic cardiomyopathy; LVNC, left ventricular noncompaction; NYHA, New York Heart Association; RCM, restrictive cardiomyopathy. (Used with permission of Elsevier from: J Am Coll Cardiol. 2014;64:304–318. https://doi.org/10.1016/j.jacc.2014.05.027, permission conveyed through Copyright Clearance Center, Inc.) [14••]

In 2014, the World Heart Federation [14••] proposed a new classification of cardiomyopathies that is modelled on that used to describe cancer (Fig. 1). The so-called MOGES system comprises five domains that provide a more flexible description of clinical phenotype combined with a detailed description of pathophysiology. The first domain (M) refers to the morphological and functional phenotype and is similar to the current system but with the addition of other ECG, imaging, structural and functional data (for example, dynamic left ventricular outflow tract obstruction and scar detected on cardiac MRI). The second descriptor (O) adds information about extracardiac disease manifestations as may occur in disease phenocopies or syndromes, and the third (G) records the family history and a multigenerational family pedigree. The (E) term captures genetic, infectious or inflammatory data, and the final domain (S) gives information on the stage of disease.

To date, the MOGES system has not been widely adopted, possibly because it is considered too complex and difficult to record in busy clinical practice. More speculatively, another barrier to its use may be its misalignment with the paradigm of modern cardiological practice that relies on a few well-established measurements such as left ventricular ejection fraction or blood pressure to target therapies. This is not necessarily the case elsewhere in medicine, most notably oncology, where a detailed understanding of molecular pathogenesis is fundamental to management.

## Changes in Therapy and the Need for Aetiological Diagnosis

Until recently, the main application of clinical genetic testing in cardiomyopathy has been the screening of families and in distinguishing rare disease phenocopies, but the development of gene replacement, RNA therapeutics and new small molecule therapies means that determination of aetiology is likely to become central to disease management. Knowledge of genotype is already incorporated into consensus guidelines for the use of implantable cardioverter defibrillators, and many targeted therapies are available or under investigation in patients with specific forms of cardiomyopathy. The examples include CRISPR/Cas9 technology to facilitate genome-editing therapy for specific diseases such as Duchenne’s muscular dystrophy and tafamidis, an effective treatment for transthyretin cardiac amyloidosis. As more aetiology-focussed treatments emerge, the need for a precise descriptions of disease phenotype will become vital in ensuring the best outcomes for individual patients.

## Evolution rather than Revolution

Based on my arguments so far, it would be reasonable to conclude that the time has finally come for the switch to a disease classification fully oriented to molecular pathology rather than the traditional descriptive clinical model. The truth, of course, is that it is impractical and perhaps impossible to create a single classification system that meets the needs of all potential users, whether they are basic scientists, clinicians or indeed patients and families. The classical cardiomyopathy subtypes still have utility, but the classification scheme needs to be modified to take into account the new phenotypes, most notably left ventricular disease characterised by myocardial scar without dilatation or even ventricular systolic impairment. In this context, use of the term “arrhythmic” as a diagnostic criterion in its own right may not be very helpful and should, I suggest, be used only in a very general sense to highlight the vital importance of ventricular arrhythmia as a clue to aetiology and as a prognostic marker across a range of clinical phenotypes.

**Fig. 2:**
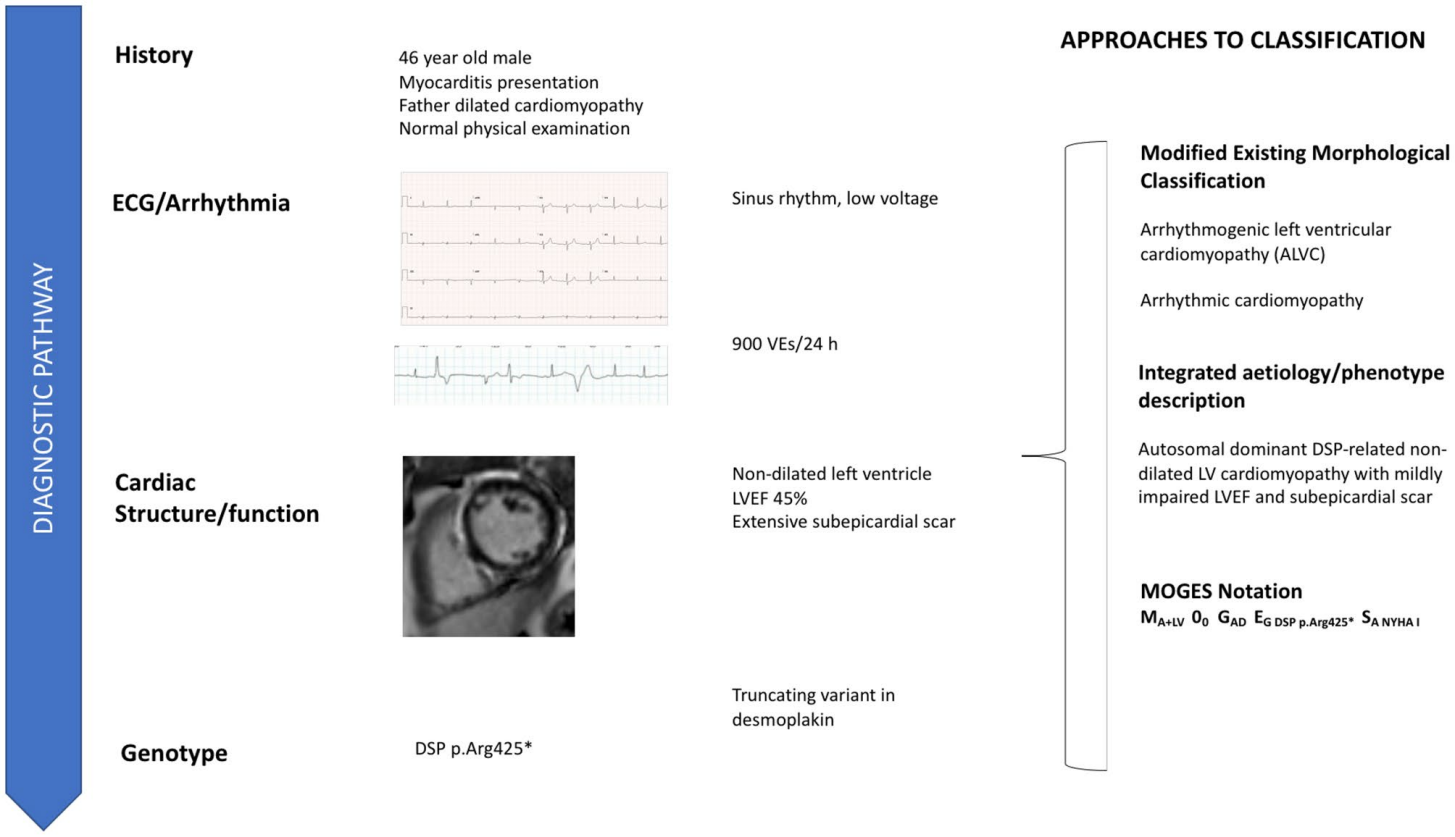
Multiparametric approach to clinical notation. This figure shows how a systematic multiparametric approach to clinical phenotyping linked with targeted diagnostics including genetic testing can be used to create highly specific phenotypes that facilitate personalised treatment plans. In this worked example, the diagnosis transforms from a simplistic categorisation to a complex genetic disorder characterised by myocardial scar and a propensity to ventricular arrhythmia. The current approach to disease nomenclature based on a single descriptor (in this case arrhythmogenic LV cardiomyopathy or even more simply arrhythmogenic cardiomyopathy) is subjective and fails to convey anything about aetiology or treatment. Key: h=hour, VE=ventricular ectopics, DSP=desmoplakin

The key to better clinical management is the integration of the morphological and functional descriptors with an extended phenotype derived from family history, ECG, tissue characterisation, circulating biomarkers and, in selected cases, tissue analysis. The very simple worked example in Fig. 2 shows how the standard tests used in everyday clinical practice can be used to provide a short but meaningful summary of a disease phenotype. The MOGES system notation is also shown as it provides a systematic notation that can be used for electronic data capture, audit and research and applies equally to familial and non-familial diseases such as myocarditis.

## Conclusions

The term cardiomyopathy continues to evolve. Advances in cardiac imaging and the introduction of genetics into everyday practice have revealed increasing complexity that poses significant challenges for diagnosis and management of people with cardiomyopathy, but the advent of new aetiology-driven therapies will drive a new approach to classification that better captures the pathophysiology of this diverse group of diseases.

## References

[1] Bridgen W. Uncommon myocardial diseases; the non-coronary cardiomyopathies. Lancet. 1957;273:1179–1184. 10.1016/s0140-6736(57)90159-9 10.1016/s0140-6736(57)90159-9 13492602

[2] Goodwin JF, Gordon H, Hollman A, Bishop MB. Clinical aspects of cardiomyopathy. Br Med J. 1961;1:69–79. 10.1136/bmj.1.5219.69 10.1136/bmj.1.5219.69 PMC1952892 13707066

[3] Report of the WHO/ISFC task force on the definition and classification of cardiomyopathies. Br Heart J. 1980;44:672–673. 10.1136/hrt.44.6.672 10.1136/hrt.44.6.672 PMC482464 7459150

[4] Richardson P, McKenna W, Bristow M, Maisch B. Mautner B, O'Connell J, et al. Report of the 1995 World Health Organization/International Society and Federation of Cardiology Task Force on the definition and classification of cardiomyopathies. Circulation. 1996;93:841–842. 10.1161/01.cir.93.5.841 10.1161/01.cir.93.5.841 8598070

[5] Elliott P, Andersson B, Arbustini E, Bilinska Z, Cecchi F, Charron P, et al. Classification of the cardiomyopathies: a position statement from the European Society Of Cardiology Working Group on Myocardial and Pericardial Diseases. Eur Heart J. 2008;29:270–276. 10.1093/eurheartj/ehm342 10.1093/eurheartj/ehm342 17916581

[6] Basso C, Corrado D, Marcus FI, Nava A, Thiene G. Arrhythmogenic right ventricular cardiomyopathy. Lancet. 2009;373:1289–1300. 10.1016/S0140-6736(09)60256-7 10.1016/S0140-6736(09)60256-7 19362677

[7] Marcus FI, McKenna WJ, Sherrill D, Basso C, Bauce B, Bluemke D, et al. Diagnosis of arrhythmogenic right ventricular cardiomyopathy/dysplasia: proposed modification of the task force criteria. Eur Heart J. 2010;31:806–814. 10.1093/eurheartj/ehq025 10.1093/eurheartj/ehq025 PMC2848326 20172912

[8] McKenna WJ, Thiene G, Nava A, Fontaliran F, Blomstrom-Lundqvist C, Fontaine G, et al. Diagnosis of arrhythmogenic right ventricular dysplasia/cardiomyopathy. Task Force of the Working Group Myocardial and Pericardial Disease of the European Society of Cardiology and of the Scientific Council on Cardiomyopathies of the International Society and Federation of Cardiology. Br Heart J. 1994;71:215–218. 10.1136/hrt.71.3.215 10.1136/hrt.71.3.215 PMC483655 8142187

[9] Corrado D, Perazzolo Marra M, Zorzi A, Beffagna G, Cipriani A, Lazzari M, et al. Diagnosis of arrhythmogenic cardiomyopathy: the Padua criteria. Int J Cardiol. 2020;319:106–114. 10.1016/j.ijcard.2020.06.005 10.1016/j.ijcard.2020.06.005 32561223

[10] Corrado D, van Tintelen PJ, McKenna WJ, Hauer R, Anastastakis A, Asimaki A, et al. Arrhythmogenic right ventricular cardiomyopathy: evaluation of the current diagnostic criteria and differential diagnosis. Eur Heart J. 2020;41:1414–1429. 10.1093/eurheartj/ehz669 10.1093/eurheartj/ehz669 PMC7138528 31637441

[11] • Elliott PM, Anastasakis A, Asimaki A, Basso C, Bauce B, Brooke MA, et al. Definition and treatment of arrhythmogenic cardiomyopathy: an updated expert panel report. Eur J Heart Fail. 2019;21:955–964. 10.1002/ejhf.1534. **Expert consensus panel that describes the conceptual framework for cardiomyopathies currently grouped under the heading of arrhythmogenic cardiomyopathies.** 10.1002/ejhf.1534 PMC6685753 31210398

[12] • Towbin JA, McKenna WJ, Abrams DJ, Ackerman MJ, Calkins H, Darrieux F, et al. 2019 HRS expert consensus statement on evaluation, risk stratification, and management of arrhythmogenic cardiomyopathy. Heart Rhythm. 2019;16:e301-e372. 10.1016/j.hrthm.2019.05.007. **This expert consensus statement aimed to provide guidance on evaluation and management of arrhythmogenic cardiomyopathy. The classification scheme illustrates the challenge of grouping very different pathologies according to the simple rubric of ventricular arrhythmia.** 10.1016/j.hrthm.2019.05.007 31078652

[13] Elliott PM (2021). Personalized medicine for dilated cardiomyopathy. Eur Heart J.

[14] •• Arbustini E, Narula N, Tavazzi L, Serio A, Grasso M, Favalli V, et al. The MOGE(S) classification of cardiomyopathy for clinicians. J Am Coll Cardiol. 2014;64:304–318. 10.1016/j.jacc.2014.05.027. **A new approach to the nosology of cardiomyopathies that may form the basis for future classification systems.** 10.1016/j.jacc.2014.05.027 25034069

